# The Breadth of Synthetic Long Peptide Vaccine-Induced CD8^+^ T Cell Responses Determines the Efficacy against Mouse Cytomegalovirus Infection

**DOI:** 10.1371/journal.ppat.1005895

**Published:** 2016-09-16

**Authors:** Eleni Panagioti, Anke Redeker, Suzanne van Duikeren, Kees LMC Franken, Jan Wouter Drijfhout, Sjoerd H. van der Burg, Ramon Arens

**Affiliations:** 1 Department of Clinical Oncology, Leiden University Medical Center, Leiden, The Netherlands; 2 Department of Immunohematology and Blood Transfusion, Leiden University Medical Center, Leiden, The Netherlands; University of North Carolina at Chapel Hill, UNITED STATES

## Abstract

There is an ultimate need for efficacious vaccines against human cytomegalovirus (HCMV), which causes severe morbidity and mortality among neonates and immunocompromised individuals. In this study we explored synthetic long peptide (SLP) vaccination as a platform modality to protect against mouse CMV (MCMV) infection in preclinical mouse models. In both C57BL/6 and BALB/c mouse strains, prime-booster vaccination with SLPs containing MHC class I restricted epitopes of MCMV resulted in the induction of strong and polyfunctional (i.e., IFN-γ^+^, TNF^+^, IL-2^+^) CD8^+^ T cell responses, equivalent in magnitude to those induced by the virus itself. SLP vaccination initially led to the formation of effector CD8^+^ T cells (KLRG1^hi^, CD44^hi^, CD127^lo^, CD62L^lo^), which eventually converted to a mixed central and effector-memory T cell phenotype. Markedly, the magnitude of the SLP vaccine-induced CD8^+^ T cell response was unrelated to the T cell functional avidity but correlated to the naive CD8^+^ T cell precursor frequency of each epitope. Vaccination with single SLPs displayed various levels of long-term protection against acute MCMV infection, but superior protection occurred after vaccination with a combination of SLPs. This finding underlines the importance of the breadth of the vaccine-induced CD8^+^ T cell response. Thus, SLP-based vaccines could be a potential strategy to prevent CMV-associated disease.

## Author Summary

The majority of infections with the betaherpesvirus human cytomegalovirus (HCMV) are clinically unnoticed, but in immunocompromised hosts HCMV infections can be severe and even fatal. Here we investigated in preclinical mouse models the efficacy and mechanisms of synthetic long peptide (SLP)-based vaccines eliciting mouse CMV (MCMV)-specific CD8^+^ T cells as a platform modality to protect against CMV infection. The percentages of MCMV-specific T cells in the circulation elicited by prime-booster SLP vaccination were equivalent or higher compared to those induced by the virus itself. We further show that the naive T cell precursor frequency rather than the functional avidity of T cells predicts the magnitude of SLP-induced CD8^+^ T cell responses. Superior protection against MCMV infection depends strongly on the combined use of distinct SLP vaccines leading to broader viral-specific responses. This finding highlights the importance of the breadth of vaccine-induced CD8^+^ T cell responses.

## Introduction

Human cytomegalovirus (HCMV) contributes substantially to morbidity in immunocompromised individuals. Organ or hematopoietic stem cell transplant recipients, people infected with HIV and patients with lymphocytic leukaemia are particularly vulnerable to HCMV-associated disease [1]. Moreover, congenital HCMV infection of unborn and new born children can lead to severe and permanent neurological symptoms [2]. Although currently available antivirals for HCMV are able to decelerate viral progression, thereby reducing the odds for major side effects, they require prolonged treatment periods and are accompanied with significant toxicity. Adoptive transfer of HCMV-specific T cells is an alternative treatment modality but is costly and laborious. The apparent burden of HCMV-associated disease and the paucity of cost-effective measures without side-effects have led to major efforts to develop effective HCMV vaccines but unfortunately no licensed vaccines are currently available [3, 4].

There is accumulating evidence that effective control of persistent viral infections requires the induction of a balanced composition of polyfunctional T cell responses [5]. T cell immunity against CMV plays a critical role in controlling the primary viral infection and latency [6]. Whereas CMV-specific CD4^+^ T cells are important during the primary infection phase, CD8^+^ T cells are associated with greater benefits at the persistent infection phase and confer superior protection during reactivation and re-exposure [7–9]. Upon CMV infection, extra-ordinary large CD8^+^ T cell responses of diverge phenotype arise. CD8^+^ T cell response kinetics specific to most antigens follow the traditional course comprised by expansion after antigen encounter, rapid contraction, long-term maintenance at low levels and acquisition of a central-memory phenotype. Interestingly, CD8^+^ T cell responses to certain CMV antigens do not dwindle post-infection but inflate and exhibit a polyfunctional effector-memory phenotype [10–13]. In immunocompromised hosts, the balance between CMV and cellular immunity is apparently underdeveloped or lost, and therefore instigating the development and/or restoration of the T cell compartment specific for CMV would be particularly informative.

The overarching aim of this study was to test a potential prophylactic vaccine platform against CMV based on synthetic long peptides (SLPs) containing immunodominant T cell epitopes. Previously, we reported that in therapeutic settings SLP-based vaccines can be successfully designed to stimulate effector and memory T cells against human papilloma virus-associated disease in mice and human [14–16]. As the efficacy of SLP-based vaccines is directly linked to the phenotypical and functional characteristics of the vaccine-induced CD8^+^ T cell response, we rigorously evaluated SLP-induced T cell responses. MCMV-specific SLP vaccines, assessed in two different mouse strains (C57BL/6 and BALB/c mice), lead to strong polyfunctional T cell responses, and combined SLP vaccines targeting different antigens provide a successful vaccine modality to control MCMV infection.

## Results

### Prime-boost vaccination with MHC class I-restricted SLPs leads to the induction of robust CD8^+^ T cell responses

To assess the potential of SLP-based vaccines in eliciting protecting CD8^+^ T cell responses against MCMV infection, we designed SLPs containing immunodominant MHC class I T cell epitopes from MCMV encoded proteins, and evaluated this vaccine platform in two different immunocompetent mouse strains with different susceptibility to MCMV; the C57BL/6 strain (MHC haplotype H-2^b^) and the more MCMV-susceptible mouse strain BALB/c (MHC haplotype H-2^d^) (S1 Table). C57BL/6 mice are less susceptible to MCMV infection compared to BALB/c mice because C57BL/6 mice express the NK cell-activating receptor Ly49H, which recognizes the MCMV protein m157 at the surface of infected cells [17–20].

**Fig 1:**
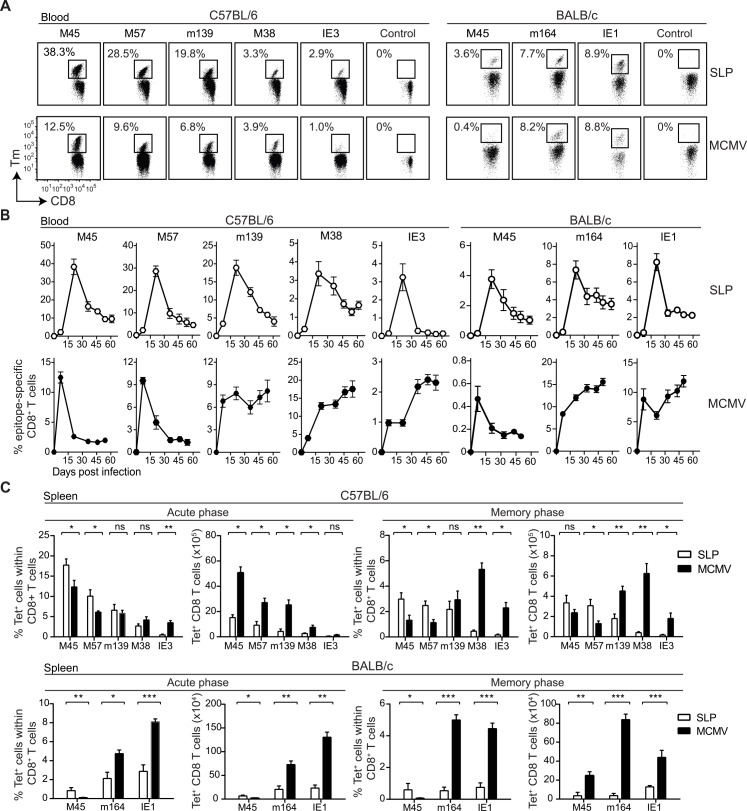
Prime-boost SLP vaccination provokes the induction of robust CD8^+^ T cell responses analogous to MCMV infection. **(A)** The magnitude of the CD8^+^ T cell responses specific to the indicated epitopes was determined in blood by MHC class I tetramer staining at day 7 post booster vaccination with SLPs and at day 7 post MCMV infection in C57BL/6 mice and in BALB/c mice. Representative flow cytometry plots show MHC class I tetramer (Tm) staining within the CD8^+^ T cell population. Numbers represent the percentage of Tm+ cells within the total CD8^+^ T cell population. **(B)** Longitudinal analysis of the epitope-specific CD8^+^ T cell responses induced by either SLP vaccination or MCMV infection in blood. Data represents mean values ± SEM (n = 6 per group). **(C)** Percentages and total numbers of splenic SLP and MCMV-specific CD8^+^ T cells during the acute phase (at day 7 post booster vaccination and day 8 and after MCMV infection) and memory phase (at day 60 post booster vaccination and day 60 post MCMV infection) are shown. Data represents mean values + SEM (n = 6 per group), and are representative of three independent experiments. *, P< 0.05; **, P<0.01; ***, P<0.001.

Mice were vaccinated subcutaneously with SLPs along with the TLR9 ligand CpG as adjuvant. The SLP vaccine administration was well tolerated without adverse events. At day 7 after SLP immunization, epitope-specific CD8^+^ T cell responses were detected in the blood but a booster vaccination was required for induction of vigorous CD8^+^ T cell responses (Fig 1A and 1B). Prime-boosting with SLP vaccines induced very high frequencies of circulating CD8^+^ T cells against the noninflationary epitopes M45_985-993_ and M57_816-824_ in C57BL/6 mice, and were even higher than the percentages of the circulating MCMV-induced CD8^+^ T cells at the peak of infection (day 7). Also the response against m139_419-426,_ known to be non-inflationary during the early phase after MCMV and at later time points as inflationary, is strong. The response against the non-inflationary M45_507-515_ epitope in BALB/c mice was even much higher in the SLP-vaccinated group as compared to the MCMV infected mice. The frequencies of the circulating CD8^+^ T cells against the inflationary M38_316-323_ and IE3_416-423_ epitopes in C57BL/6 mice and the inflationary m164_257-265_ and IE1_168-176_ epitopes in BALB/c mice were comparable (Fig 1A and 1B).

SLP vaccines containing MHC class I epitopes may comprise unidentified class II epitopes and linear B cell epitopes leading to CD4^+^ T cell and antibody responses. To exclude this possibility, we performed polychromatic intracellular cytokine staining with the SLPs and performed SLP-specific antibody ELISAs, respectively (S1 and S2 Figs). Neither MCMV-specific CD4^+^ T cells nor peptide specific Abs were detected in these assays, indicating that the designed SLPs lead exclusively to antigen-specific CD8^+^ T cell responses and that epitope-specific responses induced by SLP or MCMV can only be compared for CD8^+^ T cells.

Longitudinal analysis of the antigen-specific CD8^+^ T cell responses revealed that all SLP-induced T cell responses in both mice strains contracted gradually over time after the booster immunization (Fig 1B). Two months after the booster vaccination, the SLP-induced responses to most epitopes were still clearly detectable in blood. During MCMV infection, the epitope-specific CD8^+^ T cell responses followed a different course, consistent with previous reports [10, 11]. T cell responses to the non-inflationary epitopes M45_985-993_, M57_816-824_, and M45_507-515_ rapidly contracted after the peak response and were stably maintained in time while T cell responses to the epitopes M38_316-323_, m139_419-426_, m164_257-265_, IE1_168-176_ and IE3_416-423_ inflated (Fig 1B). These data indicate that the context of epitope expression determines the kinetics of the T cell responses, which is uniform for diverse epitopes after SLP vaccination but in the case of MCMV infection this results in a dichotomy of responses related to the chronic nature of this infection.

At the peak after the booster SLP immunization (day 7–8), high frequencies of epitope-specific CD8^+^ T cells, analogous to the responses elicited by MCMV virus were observed in the spleen (Fig 1C). However, in absolute numbers, MCMV infection led to a higher T cell magnitude compared to SLP vaccination, which can be attributed to virus-associated inflammation leading to splenomegaly. At the memory phase (day 60), MCMV-specific T cell responses to the non-inflationary epitopes were significantly lower than the equivalent SLP vaccine-induced responses (Fig 1C). The MCMV-induced CD8^+^ T cell responses to the inflationary epitopes were of higher magnitude compared to those induced by SLP vaccination.

Taken together, these results show that prime-boost vaccination with SLP vaccines containing MHC class I MCMV epitopes elicit in mouse strains with different susceptibility to MCMV high percentages of effector and memory CD8^+^ T cells that contract gradually in time.

### The T cell precursor frequency determines the magnitude of SLP vaccine-induced CD8^+^ T cell responses

Next, we aimed to dissect the underlying mechanisms of the relatively low responses to some of the SLPs (i.e. M38 and IE3 in C57BL/6; M45 in BALB/c) compared to the other. First, we endeavoured to alter the SLP sequences by altering the C-terminal cleavage, which may improve the immunogenicity (S3 Fig). However, the altered M38_316-323_ SLP did not exhibit a significant improvement in the SLP-induced T cell response whilst the altered SLP containing the IE3_416-423_ epitope elicited responses that were actually reduced.

**Fig 2:**
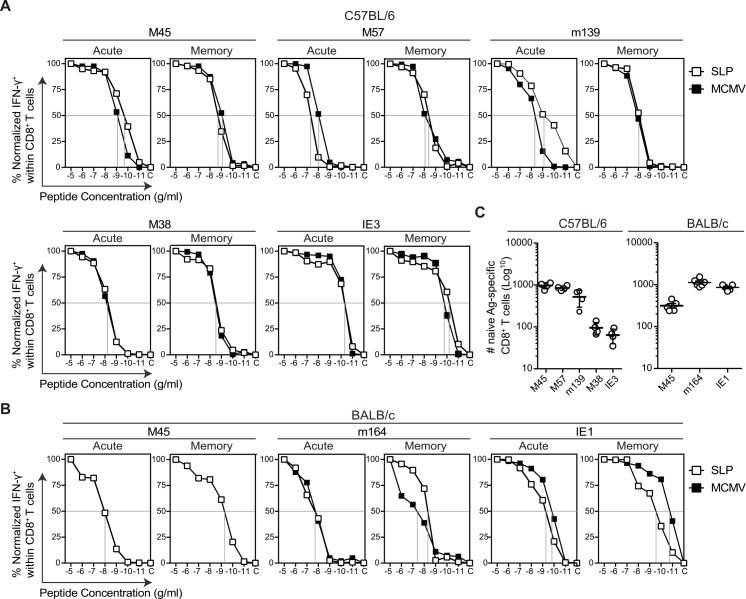
The T cell precursor frequency but not the functional avidity of the SLP vaccine-induced CD8^+^ T cells predicts the magnitude of the response. **(A)** Splenocytes from SLP vaccinated (n = 4–5 mice per epitope) and MCMV infected C57BL/6 mice (n = 4–5 mice) were isolated during the acute (day 8 post booster SLP vaccination and MCMV infection) and memory (day 60 post booster SLP vaccination and MCMV infection) phase and were restimulated with various peptide concentrations in the presence of brefeldin A. The percentage of IFN-γ producing CD8^+^ T cells was measured and normalized to the response at the highest peptide concentration. **(B)** Similar description as in (A) for experiments performed with BALB/c mice. Note that the M45_507-515_ CD8^+^ T cell response during MCMV infection was too low to obtain accurate results. Shown are the functional avidity curves and are representative of at least 2 independent experiments. Data represents mean values. **(C)** Absolute numbers of epitope specific T cell precursors present in spleen and lymph nodes of naive C57BL/6 and BALB/c mice were determined by tetramer staining. Each symbol represents an individual mouse. Data represents mean values ± SEM (n = 4–6 per group). Experiments were performed twice with similar outcome.

Then we questioned if the differences in the magnitude of the T cell responses triggered by the various single SLP vaccines might be related to the functional avidity of the T cells, which is determined by the affinity of the peptide for MHC and the TCR affinity for the peptide-MHC complex (Fig 2A and 2B). The SLPs elicited T cells with different levels of functional avidity but no correlation was found with the strength of the CD8^+^ T cell response. Moreover, in both C57BL/6 and BALB/c mice the functional avidity of the T cells, elicited either by SLP vaccines or MCMV infection, were remarkably similar and remained stable in time as they were similar during the acute and memory phase of response. Thus, differences in TCR affinity are not involved in the observed difference in the magnitude of the T cell responses.

The data presented above illustrated that factors other than peptide-MHC/TCR affinity are implicated in shaping the strength of SLP-induced T cell responses. Recently, it was shown that the precursor frequency of naive T cell populations can predict the immunodominance hierarchy of viral epitope specific CD8^+^ T cell responses [21]. To test whether the precursor frequency is predictive for the magnitude of SLP-induced T cell responses we determined the precursor frequency of all the epitopes included in this study in naive C57BL/6 and BALB/c mice (Fig 2C). In C57BL/6 mice, the precursor frequencies for the M45_985-993_ and M57_816-824_ epitopes were among the highest followed by the precursor frequencies to the m139_419-426_ epitope. The lowest precursor frequencies were detected to the M38_316-323_ and IE3_416-423_ epitopes, confirming a previous report [22]. In BALB/c mice, the highest precursor frequencies were observed for the m164_257-265_ and IE1_168-176_ epitopes whereas the frequency of M45_507-515_ specific T cells was lower (Fig 2C). Markedly, the average level of the precursor frequency of each epitope-specific CD8^+^ T cell population was proportional to the expansion of the antigen-specific populations found in mice following either SLP immunization or MCMV infection. Together, these results indicate that naive precursor frequencies rather than TCR avidity determine the magnitude of SLP vaccine-mediated CD8^+^ T cell responses.

### Phenotypical and functional characteristics of SLP-induced CD8^+^ T cells

**Fig 3:**
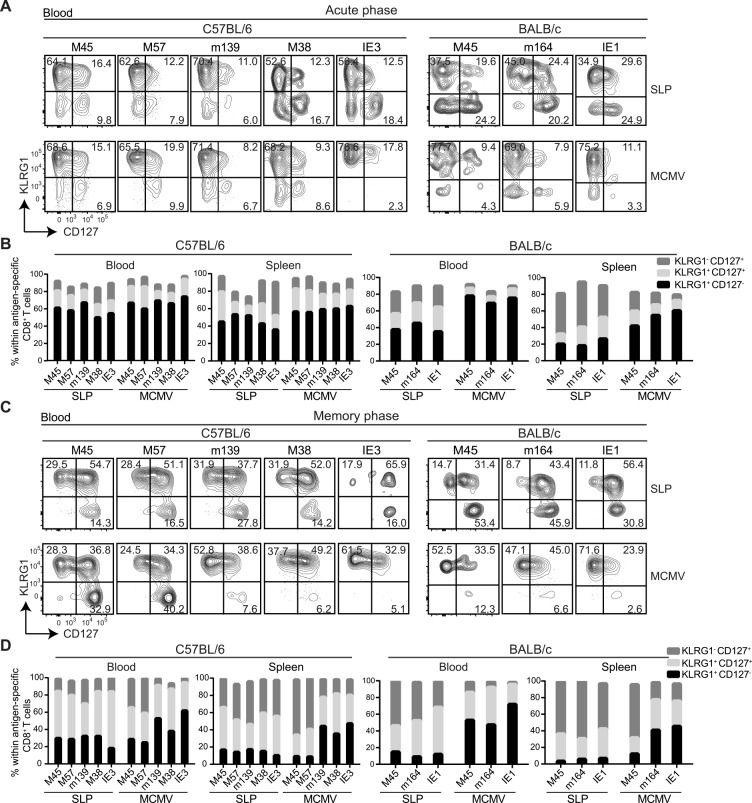
Phenotypic heterogeneity of SLP vaccine-induced CD8^+^ T cells. **(A)** Peripheral blood from either SLP immunized (day 7 after booster peptide vaccination) or MCMV infected C57BL/6 and BALB/c mice (day 7 post infection) were stained with MHC class I tetramers and for cell surface molecules. Representative plots show CD127 versus KLRG1 expression on tetramer-positive CD8^+^ T cells. **(B)** Cell-surface characteristics of tetramer-positive CD8^+^ T cells in blood and spleen at day 8 post booster SLP vaccination and day 8 after MCMV infection. **(C)** Peripheral blood from either SLP immunized (day 60 after booster peptide vaccination) or MCMV infected C57BL/6 and BALB/c mice (day 60 post infection) were stained with MHC class I tetramers and for cell surface molecules. Representative plots show CD127 versus KLRG1 expression on tetramer-positive CD8^+^ T cells. **(D)** Cell-surface characteristics of antigen-specific CD8^+^ T cells in blood and spleen at day 60 post booster SLP vaccination and day 60 after MCMV infection. Data represents mean values, and are representative of three independent experiments (n = 6 per group).

To assess the phenotypical and functional quality of MCMV-specific CD8^+^ T cells induced by either the SLPs or the virus, we determined the formation of the diverse T cell subsets that develop after antigenic challenge. Early after the booster, SLP vaccination resulted in the induction of a highly activated CD8^+^ T cell subset exhibiting an effector-like phenotype (CD62L^lo^, CD44^hi^, CD127^lo^, KLRG1^hi^), which completely resembled the MCMV-specific T cell phenotype during the acute phase of the infection (Fig 3A and 3B). In the memory phase, both SLP- and MCMV-induced T cell phenotypic traits diverged (Fig 3C and 3D). All SLP-induced CD8^+^ T cells exhibited a fairly mixed phenotype sharing features of both central-memory T cells (CD62L^hi^, CD44^lo^, CD127^hi^, KLRG1^lo^), effector-memory T cells (KLRG1^hi^, CD44^hi^, CD127^lo^, CD62L^lo^) but also an intermediate phenotype (i.e. KLRG1^hi^, CD127^hi^). As expected, during MCMV infection, the non-inflationary M45_985-993,_ M45_507-515_ and M57_816-824_-specific CD8^+^ T cells gained a predominant central memory-like phenotype while the inflationary M38_316-323_, m139_419-426_, IE3_416-423_, m164_257-265_ and IE1_168-176_-specific T cells appeared mostly effector-memory like.

**Fig 4:**
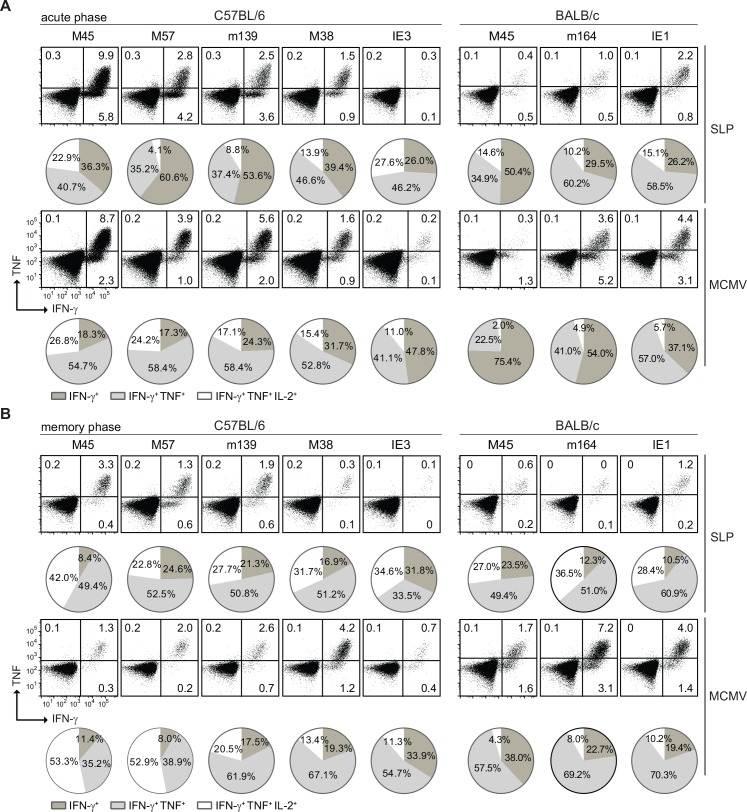
SLP vaccination elicits polyfunctional CD8^+^ T cells. Following SLP vaccination or MCMV infection the cytokine polyfunctionality of splenic CD8^+^ T cells was determined after peptide restimulation. Representative plots show IFN-γ versus TNF production at **(A)** day 8 (acute phase) and **(B)** day 60 (memory phase) post booster vaccination and post MCMV infection. Pie charts depict the percentages of the single (IFN-γ), double (IFN-γ/TNF) and triple (IFN-γ/TNF/IL-2) cytokine producers of each antigen-specific T cell population upon peptide stimulation. Data represents mean values, and are representative of three independent experiments (n = 4–5 per group). Statistics of the results depicted in these pie charts are reported in S4 Fig.

To assess the cytokine profiles of the SLP-induced CD8^+^ T cells, we performed intracellular cytokine staining for IFN-γ, TNF and IL-2 and compared these to MCMV-induced T cells. At the peak response after booster vaccination, SLP-induced T cells consisted mainly of single IFN-γ and double IFN-γ/TNF producing populations (Figs 4A and S4). The cytokine producing traits of the MCMV-induced effector CD8^+^ T cells matched in general with the SLP-elicited T cells. Except relatively more single IFN-γ producing CD8^+^ T cells after MCMV infection compared to SLP vaccination were found in the T cell populations reactive to the epitopes IE3_416-423_, IE1_168-176_, M45_507-515_ and m164_257-265._

At the memory phase, the SLP-specific CD8^+^ T cells gained the ability to co-produce the three cytokines, at the expense of single cytokine producing cells (Figs 4B and S4). This gain in triple cytokine production (IFN-γ/TNF/IL-2) during MCMV infection is mainly observed in the non-inflationary CD8^+^ T cells. Both during the acute and memory phase, the percentage of the total CD8^+^ T cell population producing IFN-γ, either in case of SLP vaccination or MCMV infection, corresponded to the percentage of MHC class I tetramers, indicating full differentiation of the elicited T cells.

**Fig 5:**
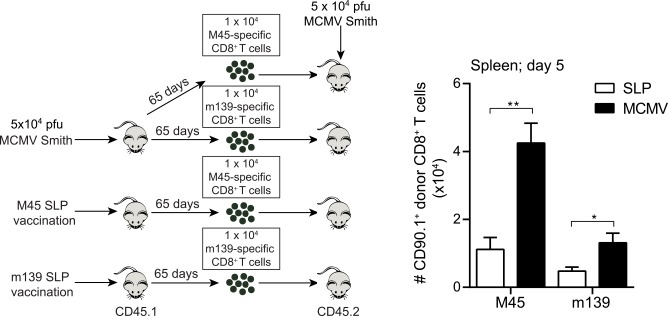
Secondary expansion potential of SLP-induced CD8^+^ T cells upon MCMV challenge. M45_985-993_ and m139_419-426_ epitope specific CD8^+^ T cells were isolated from the spleen at day 60 after booster vaccination and infection of SLP vaccinated and MCMV infected CD45.1 mice. 1 × 10^4^ antigen-specific CD8^+^ T cells were adoptively transferred into naive C57BL/6 (CD45.2) recipient mice. Recipient mice were subsequently infected i.p. with 5 × 10^4^ PFU MCMV-Smith. The total numbers of the donor derived M45_985-993_ and m139_419-426_ CD8^+^ T cells were determined in the spleen at day 5 post challenge. Data represent mean values + SEM (n = 5). Experiments were performed twice with similar outcome. *, P<0.05; **, P<0.01.

A hallmark of memory T cells is the ability to undergo secondary expansion upon antigenic challenge [23]. To assess this property of vaccine-induced memory T cells, we performed adoptive transfer experiments in which congenically marked (CD45.1^+^) memory M45_985-993_ and m139_419-426_-specific CD8^+^ T cells from SLP vaccinated and MCMV infected mice were isolated and transferred into naive recipient mice, which were subsequently challenged with MCMV (Fig 5). SLP-induced M45_985-993_ and m139_419-426_-specific T cells expanded; albeit to a lesser extend as compared to the MCMV-induced (Fig 5). The MCMV-elicited M45_985-993_-specific T cells exhibited, corresponding to their central-memory phenotype, a superior capacity in expansion as compared to the MCMV-elicited m139_419-426_-specific T cells with an effector-memory phenotype. Of note, the expansion of the SLP-induced M45-specific T cells was comparable to the m139-specific T cells induced by MCMV, although the phenotype of SLP-induced cells were more central-memory like. This indicates that the instruction that T cells receive in different settings can result in cells with a different expansion potential despite a seemingly similar phenotype based on markers for central/effector memory cells. All together we conclude that SLP-based vaccines induce a heterogeneous pool of memory T cells with a secondary expansion potential that is somewhat lower as compared to memory T cells elicited by virulent virus.

### Combinatorial SLP vaccination confers superior efficacy against CMV infection

**Fig 6:**
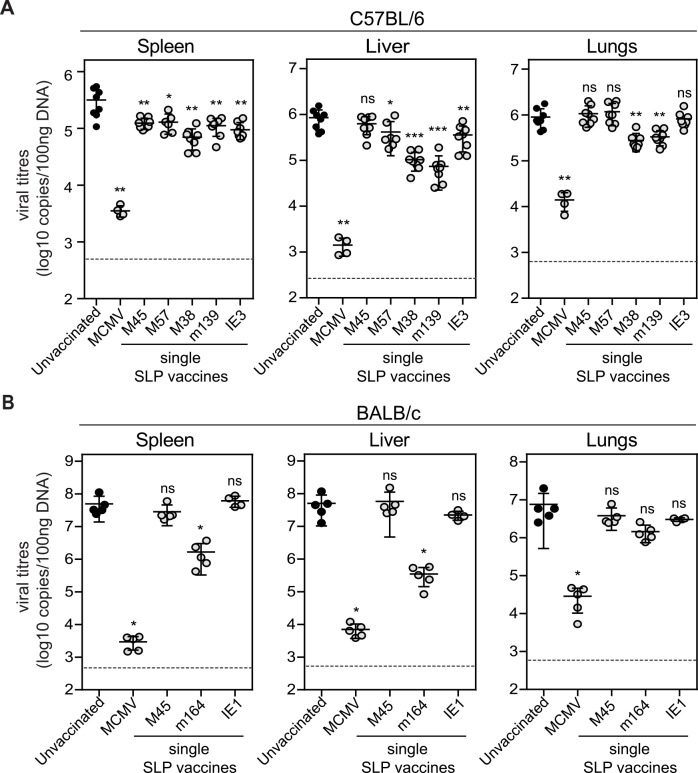
Efficacy of single SLP vaccines against acute MCMV infection. Unvaccinated (naive), SLP vaccinated and MCMV infected C57BL/6 and BALB/c mice were challenged at day 60 post vaccination/infection with 5 × 10^4^ PFU and 5 × 10^3^ PFU salivary gland-derived MCMV Smith, respectively. At day 5 post challenge, spleen, liver, and lungs were isolated and the viral genome copies were determined by qPCR. The viral titres of individual **(A)** C57BL/6 and **(B)** BALB/c mice are depicted (n = 5–8 per group). Mean ± SEM is indicated. Dashed line represents the detection limit as measured in naive mice. Experiments were performed twice with similar outcome. *, P< 0.05; **, P<0.01; ***, P<0.001; ns, not significant.

The various SLP vaccine formulations were evaluated for their capacity to confer protection against MCMV challenge (at day 60 after booster vaccination). In C57BL/6 mice, the viral load of unvaccinated (naive) mice challenged with MCMV was found to be significantly higher in spleen, liver and lungs, when compared to the viral load of MCMV re-challenged mice that successfully controlled a previous MCMV infection, indicating that pre-existing immunity to MCMV can clearly reduce the viral load upon re-infection (Fig 6A). All the different SLP vaccines resulted in a reduction in viral load in the spleen compared to unvaccinated mice, albeit less effective when compared to MCMV infected mice. Mice vaccinated with the SLPs containing the M38_316-323_ and m139_419-426_ epitopes display a significant reduction in viral titres in the liver and lungs. Also, the M57_816-824_ and IE3_416-423_ epitope containing SLPs were capable in reducing the viral replication in the liver after MCMV challenge, albeit to a lesser extent (Fig 6A).

Re-challenge of MCMV infected BALB/c mice resulted in substantial protection of the m164_257-265_ epitope containing SLP vaccine in spleen and liver (Fig 6B). The M45_507-515_ and IE1_168-176_ epitope containing SLPs however did not induce protective immunity in vaccinated mice. These results indicate that certain SLPs but not all have the potency to elicit protective immunity against virus challenge, and that this protection is not necessarily correlating to the size of the SLP-induced CD8^+^ T cell response.

**Fig 7:**
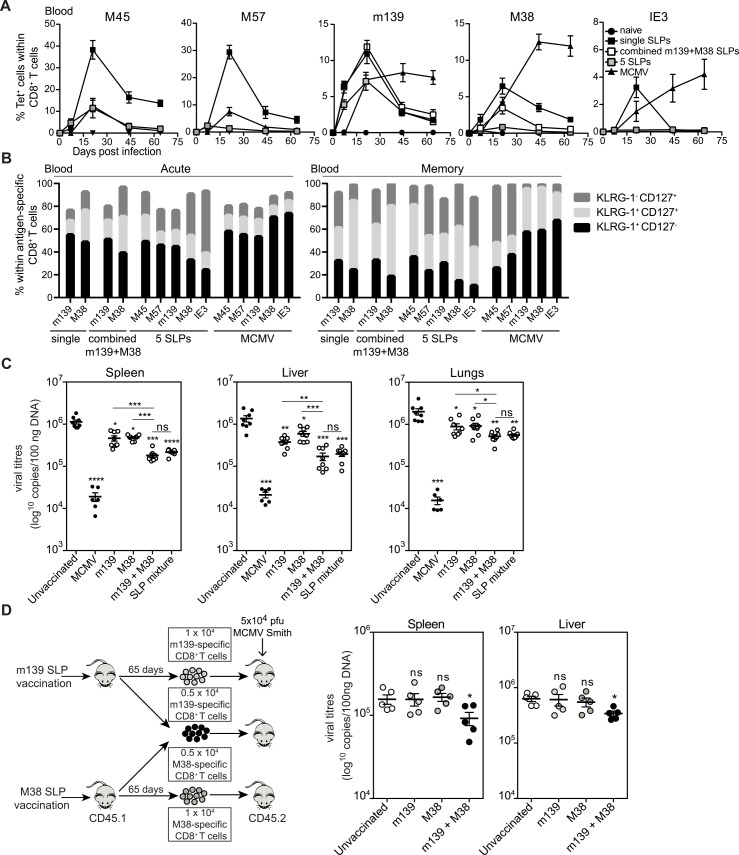
Enhanced efficacy against CMV infection by combinatorial use of distinct SLP vaccines. C57BL/6 mice were vaccinated with single m139 and M38 SLPs, a combination of these SLPs, and a mixture of five different (M45, M57, m139, M38, IE3) SLPs. **(A)** Kinetics of the antigen-specific CD8^+^ T cells in the blood. Data shown are mean values ± SEM (n = 8) **(B)** Phenotypic profile of the (combinatorial) SLP vaccine-induced CD8^+^ T cells in blood at day 7 (acute) and day 60 (memory) post booster vaccination. Data represent mean values (n = 8). Experiment was performed twice with similar outcome. **(C)** Unvaccinated (naive), (combined) SLP vaccinated and MCMV infected C57BL/6 mice were challenged 65 day post booster-vaccination/infection with 5 × 10^4^ PFU salivary gland-derived MCMV Smith. At day 5 post challenge, liver, lungs and spleen were isolated and the viral genome copies were determined by qPCR. The viral titres of individual mice are depicted (n = 6–8 per group). **(D)** Splenic antigen-specific CD8^+^ T cells were sorted at day 60 post booster-vaccination of single m139_419-426_ or M38_316-323_ SLP vaccinated CD45.1 mice. 1 × 10^4^ m139_419-426_, 1 × 10^4^ M38_316-323_ or 0.5 × 10^4^ m139_419-426_ plus 0.5 × 10^4^ M38_316-323_ CD8^+^ T cells were adoptively transferred into naive CD45.2 recipient mice. Subsequently, recipient mice were infected i.p. with 5 × 10^4^ PFU MCMV-Smith. At day 5 post infection spleen and liver were isolated and the viral genome copies were determined by qPCR. The viral titres of individual mice are depicted. Mean ± SEM is also shown. The detection limit was below 1000 genome copies as measured in naive mice. Experiments were performed twice with similar outcome. Statistical difference is indicated (*, P<0.05; **, P<0.01; ***, P<0.001; ****, P<0.0001; ns, not significant) as compared to the unvaccinated group unless otherwise indicated.

Since vaccination with the m139_419-426_ and M38_316-323_ epitope containing SLPs was accompanied with some reduction of the viral load, we examined in C57BL/6 mice whether vaccination with these two, or even more, SLPs combined is able to exceed the protection efficacy of single SLP immunization. Strong and long-lived peptide-specific CD8^+^ T cell responses were measured in mice vaccinated with the mixture of the m139 SLP plus the M38 SLP and with a mixture of all 5 SLP vaccines (Fig 7A). Notably, the T cell response against each peptide epitope with the combined SLP vaccines was lower as compared to single SLP vaccination (except for the m139-specific response), indicating that competition among antigen-specific CD8^+^ T cell populations can occur in multivalent vaccines. Especially, altered were the responses to the epitopes in M57 and IE3 because these were not boosted (Fig 7A). Such competition among T cells during boosting has also been observed after viral infection [24]. The kinetics of the combined SLP vaccine-induced T cell responses was found to be similar to single SLP vaccines, and the phenotype (Fig 7B) and cytokine polyfunctionality of the T cells as well (S5 Fig).

At day 60 post booster vaccination, mice were challenged with MCMV and 5 days later viral titres were measured in different organ tissues. The efficacy of the combined SLPs to protect upon acute MCMV challenge was remarkably improved compared to the single SLP vaccines, as all mice that received a mixed SLP vaccine exhibited significant reduction in the viral load, especially in the liver (Fig 7C), suggesting that the breadth of the response or the magnitude of the total anti-viral response is important. Remarkably, the combination of the m139 SLP with the M38 SLP was as efficacious as the combination with all 5 SLPs. To assess if superior viral control was related to the breadth of the response, we adoptively transferred 1 × 10^4^ m139 SLP-induced CD8^+^ T cells, 1 × 10^4^ M38 SLP-induced CD8^+^ T cells, or an equal total number of a pool of both m139 (0.5 × 10^4^) and M38 (0.5 × 10^4^) SLP-induced CD8^+^ T cells in naive recipient mice (Fig 7D). The transfer of SLP-induced CD8^+^ T cell populations with a dual specificity resulted in a significant reduction in viral titres, while the transfers of equal amounts of T cells with single specificity did not. Thus, combinations of at least two distinct SLP vaccines have an increased potency to protect compared to single SLP vaccines, indicating that the breadth of the vaccine-induced CD8^+^ T cell responses plays a crucial role in anti-viral immunity. We conclude that vaccination with single SLPs can be applied as a prophylactic vaccine strategy against CMV infection, but vaccination with combinations of different SLPs serve as a superior vaccine technology platform against viral challenge.

## Discussion

In this study we report that SLP-based vaccines are an effective modality against CMV infection. In a prime-boost vaccine regimen, SLPs containing single MCMV epitopes are highly immunogenic in both C57BL/6 and BALB/c mice, and generate long-lasting polyfunctional CD8^+^ T cell responses. Our study revealed three key findings. First, the magnitude and phenotype of the SLP-induced T cell responses initially resemble those evoked by a real viral infection. Second, the magnitude of the SLP-induced T cell response strongly correlated to the naive T cell precursor frequency, and third the protection against viral infection by SLP-induced memory CD8^+^ T cells was most pronounced when vaccination was performed with combinations of distinct SLPs leading to an increased breadth of the antigen-specific T cell response.

In the last decades many vaccine strategies such as attenuated virus, DNA constructs, protein, and virally vectored vaccines targeting HCMV have been developed [3, 4]. The focus of most of these vaccines was to generate protective antibodies. Our finding that SLP-based vaccines that solely provoke CD8^+^ T cell responses are efficacious suggests that the design of more efficient vaccines against CMV should incorporate the induction of CD8^+^ T cell immunity. Although we observed some epitope competition among SLP vaccine-induced CD8^+^ T cell responses, we anticipate that inclusion of CD4^+^ T cell and B cell epitopes will further improve the vaccine efficacy given that CD4^+^ T cells and antibodies have also antiviral actions against CMV. Moreover, SLP-based vaccines allow further refinement by different prime-booster regimens and by combinations with adjuvants, immunomodulatory antibodies or other vaccine platforms [25]. Conceivably, this will positively impact the phenotype and effectivity of the vaccine-induced T cells.

As to date, the high CD8^+^ T cells responses elicited with the SLP vaccines encoding MCMV epitopes have not been observed before with other SLPs including those containing epitopes of human papilloma virus (HPV) [14], lymphocytic choriomeningitis virus (LCMV) [26], influenza [27] or model antigens [28]. This may be explained by the relatively high precursor frequency of T cells responding to some of the MCMV epitopes. Our study indicates that it is of interest for T cell-based vaccines to determine the antigen-specific T cell precursor frequencies as these correlate to the magnitude of the vaccine-induced antigen-specific response, allowing the selection of epitopes generating the most robust responses. This knowledge can be very useful for development of vaccines that are based on selection of epitopes. Nevertheless, the magnitude of the vaccine-induced T cell response appears not necessarily to correlate to protective immunity but seems to depend also on the specificity. For example, in C57BL/6 mice, the large vaccine-elicited responses to the M45_985–993_ and M57_816-824_ epitopes do not provide as good protection as the seemingly lower response to the M38_316–323_ epitope. Similarly, in BALB/c mice the m164 SLP confers immunity in liver and spleen whereas the IE1_168-176_ epitope containing SLP, which is analogous in magnitude, does not show protective effects. Previous studies using short peptide or DNA vaccination also reported that the strength of the vaccine-induced IE1-specific CD8^+^ T cell response does not necessarily correlate to protection [29–31], suggesting that the quality of the vaccine-induced T cell is more decisive.

Dissimilarities in transcription of viral genes [32], which may even vary in different tissues, as well as the efficiency of peptide processing and presentation at the cell surface, may also be implicated in the differential efficacy of the T cell response to each particular epitope to confer resistance to MCMV. In this respect it is of interest to note that SLP vaccines containing “inflationary” epitopes (i.e., M38 and m139) elicit better protection as compared to the non-inflationary epitopes. This may relate to differences in the presentation of the inflationary epitopes as compared to the non-inflationary epitopes by infected cells and/or by (cross-presenting) APCs.

An important requirement for memory inflation is chronic antigenic exposure [12]. The fact that SLPs do not elicit inflation suggests that SLPs are broken down in such a manner that epitopes are not presented over a long period of time as occurs during persistent CMV infection. Other factors important for memory inflation during CMV infection, such as dependence on certain T cell costimulatory interactions (e.g., CD27-CD70 [33]), are likely also not in place at late time points post SLP vaccination. In addition, a characteristic feature of inflationary T cells is their predominant effector-memory like phenotype. The SLP vaccine-induced T cells are not mostly effector-memory like, as may be expected because of the apparent absence of memory inflation. Although the expansion of the SLP-induced CD8^+^ T cells seems to be somewhat negatively influenced as compared to virus-induced T cells, it remains to be determined whether protection on a per-cell basis is influenced as well. Nevertheless, the SLP-induced T cells were well capable to reduce the viral load upon viral challenge, especially when a mixture of distinct SLPs was used for vaccination. The somewhat lesser expansion potential of the SLP-induced T cells might relate to some of the differences in the phenotype of the SLP and MCMV-elicited T cells. Although the effector T cells induced by either SLP boost vaccination or MCMV infection had an analogous phenotype (KLRG1^hi^, CD44^hi^, CD127^low^, CD62L^low^, IL2^+/-^) and cytokine profile, the memory T cells elicited by SLPs displayed a mixed profile of effector-memory (KLRG1^hi^, CD127^lo^), central-memory (KLRG1^lo^, CD127^hi^) and double-positive T cells (KLRG1^hi^, CD127^hi^). In contrast, MCMV infection induces a more polarized phenotype: either a central-memory phenotype (mainly non-inflationary responses) or an effector-memory phenotype (mainly inflationary responses). Whether a lack of CD4^+^ T cell helper signals [34] or a lack of virus-associated inflammatory signals [26] is responsible for the observed SLP vaccine-associated phenotype and secondary expansion potential remains to be examined in future studies.

We showed that the efficacy of SLP vaccines to protect against MCMV is primarily driven by the breadth of the CD8^+^ T cell responses rather than the magnitude of the individual SLP vaccine-induced T cell responses. A possible explanation is that viral infected cells are to a certain degree resistant to CD8^+^ T cell mediated killing due to sophisticated immune evasion mechanisms including downmodulation of MHC class I molecules and prevention of apoptosis [35–37]. Accordingly, it has been estimated that one effector CD8^+^ T cell kills only 2–16 MCMV-infected cells per day and the probability of death of infected cells increases for those contacted by more than two CTLs, which is indicative of CTL cooperation [38]. Our study suggests that multiple encounters with cytotoxic CD8^+^ T cells with different specificity result in more effective killing of infected cells.

Overall, this study provided evidence that SLP-based vaccines eliciting memory CD8^+^ T cell responses have protective effects against acute MCMV infection with respect to lowering the viral load in tissues. These promising results highlight the need for additional studies to elucidate the role of vaccine-induced T cells against CMV and other persistent viral infections.

## Materials and Methods

### Mice

C57BL/6 mice and BALB/c mice were purchased from Charles River Laboratories (L'Arbresle, France). CD45.1 (Ly5.1) congenic mice on a C57BL/6 background were obtained from The Jackson Laboratory. Mice were maintained under specific-pathogen-free conditions at the Central Animal Facility of Leiden University Medical Center (LUMC), and were aged 8–10 weeks at the beginning of each experiment. The mice did not undergo any immunosuppressive treatments and were fully immunocompetent.

### Ethics statement

All animal experimental protocols were approved by the LUMC Animal Experiments Ethical Committee in accordance with the Dutch Experiments on Animals Act and the Council of Europe (numbers 13156 and 14187).

### Virus production, infections and determination of viral load

MCMV virus stocks were prepared from salivary glands of BALB/c mice infected with MCMV-Smith (American Type Culture Collection (ATCC)). The viral titres of the produced virus stocks were determined by viral plaque assays with 3T3 mouse embryonic fibroblasts (MEFs) (ATCC). Age- and gender-matched C57BL/6 mice were infected with 5 × 10^4^ PFU MCMV, and age- and gender-matched BALB/c mice with 5 × 10^3^ PFU MCMV. Viruses were administered intraperitoneally (i.p) in a total volume of 400 μl in PBS. At 65 days post-booster vaccination or infection, SLP vaccinated or MCMV infected mice were (re)-challenged with 5 × 10^4^ PFU MCMV. Determination of viral load was performed by real-time PCR as described previously [39].

### Peptides synthesis and vaccination

Short (9–10 aa) and long (20–21 aa) peptides containing MHC class I-restricted T cell epitopes from MCMV encoded proteins in C57BL/6 and BALB/c mice were produced at the peptide facility of the LUMC (peptide sequences are described in S1 Table). The purity of the synthesized peptides (75–90%) was determined by HPLC and the molecular weight by mass spectrometry. Synthetic long peptide (SLP) vaccinations were administered subcutaneously (s.c.) at the tail base by delivery of 50 μg SLP and 20 μg CpG (ODN 1826, InvivoGen) dissolved in PBS in a total volume of 50 μl. Booster SLP vaccinations were provided after 2 weeks. Vaccination with a mixture of SLPs was done with 50 μg of each SLP and 20 μg CpG.

### Flow cytometry

Cell surface and intracellular cytokine stainings of splenocytes and blood lymphocytes were performed as described [40]. For examination of intracellular cytokine production, single cell suspensions were stimulated with short peptides for 5 h in the presence of brefeldin A or with long peptides for 8 h of which the last 6 h in presence of brefeldin A (Golgiplug; BD Pharmingen). MHC class I tetramers specific for the following MCMV epitopes: M45_985–993_, M57_816–824_, m139_419-426_, M38_316–323_, and IE3_416–423_ in C57BL/6 mice and M45_507–515_, m164_257-265_ and IE1_168-176_ in BALB/c mice were produced as reported [41]. Fluorochrome-conjugated mAbs were purchased from BD Biosciences, Biolegend or eBioscience. Flow cytometry gating strategies are shown in S6 Fig. Samples were acquired with the LSRFortessa cytometer (BD Biosciences) and analysed with FlowJo-V10 software (Tree star).

### T cell functional avidity assay

A peptide dose-response titration was performed to determine and compare the TCR avidity of the CD8^+^ T cells induced after SLP vaccination and MCMV infection at the acute and memory phase. In brief, splenocytes were stimulated with various concentrations of short peptide in presence of 2 μg/ml brefeldin A for 5 h at 37°C. Subsequently, cell surface staining and an intracellular IFN-γ staining were performed. Responses were analysed using the same approach as described above.

### Antibody detection by ELISA

Blood was collected from the retro-orbital plexus and after brief centrifugation, sera were obtained and stored at −20°C. Specific immunoglobulin levels in serum were measured by ELISA as described [39]. Briefly, Nunc-Immuno Maxisorp plates (Fisher Scientific) were coated either with 2 μg/ml SLPs or with MCMV-Smith in bicarbonate buffer, and after blocking with skim milk powder (Fluka BioChemika) diluted sera were added. Next, plates were incubated with HRP-conjugated antibodies (SouthernBiotech) to detect different Ab isotypes. Plates were developed with TMB substrate (Sigma Aldrich) and the colour reaction was stopped by addition of 1M H_2_SO_4_. To serve as a positive control, a peptide from the M2 protein (eM2) of influenza A virus with identified ability to induce antibodies and corresponding serum was used. Optical density was read at 450 nm (OD_450_) using a Microplate reader (Model 680, Bio-Rad).

### Determination of the T cell precursor frequency

To determine the endogenous naive precursor frequency of MCMV-specific CD8^+^ T cell populations in C57BL/6 and BALB/c mice, enrichment assays of antigen-specific CD8^+^ T cells were performed as described [42]. In short, single cell suspensions were generated from pooled spleen and lymph nodes (mesenteric, inguinal, cervical, axillary, and brachial) of individual mice. Cells were stained with PE and APC-labelled MHC class I tetramers for 0.5 h at RT, then washed, labelled with anti-PE and anti-APC microbeads (Miltenyi Biotec), and passed over a magnetized LS column (Miltenyi Biotec). The tetramer-enriched fractions were stained with fluorochrome labelled Abs against CD3 (clone 500A2), CD4 (clone L3T4), CD8 (clone 53–6.7) for 30 min at 4°C, and subsequently analysed. Samples were acquired with the LSRFortessa cytometer (BD Biosciences).

### Adoptive transfers

The expansion capacity and vaccine efficacy of SLP vaccine and/or MCMV-induced antigen-specific CD8^+^ T cells was determined by adoptive transfers. Splenic CD8^+^ T cells from chronically (day 60) infected and SLP vaccinated CD45.1^+^ mice were enriched with magnetic sorting using the CD8^+^ T cell isolation kit in accordance with the manufacturer’s protocol (Miltenyi Biotec). Next, cells were stained with MHC class I tetramers and with fluorochrome labelled antibodies against CD3 and CD8. Tetramer positive CD8^+^ T cells were sorted using a FACSAria II Cell Sorter (BD Biosciences) and 1 × 10^4^ tetramer^+^ CD8^+^ T cells were transferred (retro-orbital in a total volume of 200μl in PBS) into naive CD45.2^+^ C57BL/6 recipients. Recipients were subsequently (2 h later) infected with 5 × 10^4^ PFU MCMV. At day 5 post viral challenge the viral titres were determined by qPCR and the number of donor-specific CD8^+^ T cells by flow cytometry.

### Statistical analyses

Statistical significance was assessed with Student’s t-test or ANOVA using GraphPad Prism software (GraphPad Software Inc., USA). The level of statistical significance was set at P<0.05.

## Supporting Information

S1 Table Depiction of the MHC class I peptide epitopes and SLPs. (DOCX)

S1 Fig MHC class I SLP vaccines do not elicit activation of CD4^+^ T cells. **(A)** C57BL/6 and (B) BALB/c mice were vaccinated SLPs, and at day 8 following vaccination the intracellular cytokine production by CD8^+^ and CD4^+^ T cells was determined after restimulation with long peptide. Representative plots show IFN-γ versus TNF production of CD8^+^ and CD4^+^ T cells of **(A)** M57_816-824_ and IE3_416-423_ epitope containing SLP vaccinated C57BL/6 mice, and of **(B)** m164_257-265_ and IE1_168-176_ epitope containing SLP-vaccinated BALB/c mice. No peptide controls and naive mice were used as negative controls. IFN-γ and TNF reactivity was only observed in CD8^+^ T cells. Similar data were observed for the other SLPs (i.e., M45, m139 and M38 SLPs in C57BL/6 mice and M45 SLP in BALB/c mice). Experiments were performed twice with similar outcome (n = 3–4 mice per group).
(EPS)

S2 Fig Vaccination with MHC class I restricted SLPs does not lead to the induction of peptide-specific Abs. At day 60 post booster SLP vaccination or MCMV infection serum samples were obtained from C57BL/6 and BALB/c mice. ELISA plates were coated with either SLP **(A, C)** or MCMV **(B, D)** and detection of peptide specific Ab (including IgG, various IgG subtypes, and IgE) in the serum was assessed. To serve as a positive control, a peptide with known ability to induce antibodies and corresponding serum was used. Optical density values were measured at 450nm. Each symbol represents an individual mouse. Mean value ± SEM (n = 3–4 mice per group) is indicated. Experiments were performed twice with similar outcome.
(EPS)

S3 Fig Epitope alteration at the C-terminus did not improve the immunogenicity. Alterations of the C-terminus sequence residues of the M38_316-323_ and IE3_416-423_ epitope containing SLPs was performed and their capacity to induce epitope-specific CD8^+^ T cell responses was tested and compared to the unaltered SLPs. MHC class I tetramer staining on blood was performed to assess the immunogenicity of the M38_316-323_ and IE3_416-423_ peptides on C57BL/6 mice (n = 4). Data represent mean values ± SEM (n = 4 mice/group). *, P<0.05; **, P<0.01; ns, not significant.
(EPS)

S4 Fig SLP vaccination elicits polyfunctional CD8^+^ T cells. Statistical analysis of the results depicted in the pie charts of Fig 4. The cytokine polyfunctionality of the splenic CD8^+^ T cells elicited after SLP vaccination or MCMV infection was determined after 5 hours *in vitro* restimulation with short peptides. Graphs show the percentages of the single (IFN-γ), double (IFN-γ/TNF) and triple (IFN-γ/TNF/IL-2) cytokine producers of each antigen-specific T cell population upon peptide stimulation. Data represents mean values + SEM, and are representative of three independent experiments (n = 4–5 per group). *, P<0.05; **, P<0.01; ***, P<0.001; ****, P<0.0001; ns, not significant.
(EPS)

S5 Fig Analysis of the magnitude and functionality of the multivalent SLP vaccine-induced CD8^+^ T cells. C57BL/6 mice were prime-boost vaccinated with a mixture of 5 SLPs. The cytokine production capacity of the splenic SLP vaccine-induced CD8^+^ T cells was examined by intracellular cytokine staining at day 8 (acute phase) and 60 (memory phase) after booster vaccination. Pie charts depict the percentages of the single (IFN-γ), double (IFN-γ/TNF) and triple (IFN-γ/TNF/IL-2) cytokine producers of each antigen-specific T cell population after 5 hours *in vitro* restimulation with short peptide. Data represents mean values, and are representative of three independent experiments (n = 6 mice per group).
(EPS)

S6 Fig Flow cytometry gating strategy. **(A)** Representative plots show the gating strategy to detect MHC class I tetramer positive cells in blood and spleen. In sequential gating, cells were first gated on lymphocytes (forward scatter vs. side scatter), then singlets (FSC-A vs. FSC-H), followed by viability (FSC-A vs. 7-AAD). Next, live cells were analysed for the expression of CD3 and CD8 while cells positive for CD4 were excluded. MHC class I tetramer positive cells (CD8 vs. Tm) were analysed for the expression of KLRG1, CD44, CD127 and CD62L surface markers. **(B)** Gating strategy for intracellular cytokine staining. Utilizing a sequential gating analysis, cells were initially gated on lymphocytes (FCS-A vs. SCS-A) and singlets (FCS-A vs. FSC-H). Next, cells were analysed for the expression of CD3 and CD8 while cells positive for CD4 were excluded. IFN-γ producing CD8^+^ T cells were further analysed for expression of TNF and IL-2.
(EPS)
