# Supplementary material for: The Breadth of Synthetic Long Peptide Vaccine-Induced CD8+ T Cell Responses Determines the Efficacy against Mouse Cytomegalovirus Infection
Source: PLoS Pathog. 2016 Sep 16;12(9):e1005895. doi: 10.1371/journal.ppat.1005895 (PMC5026341; doi:10.1371/journal.ppat.1005895)
Supplement: S1 Table — (DOCX) [file ppat.1005895.s001.docx]

**Table 1. MCMV peptide-specific CD8 T cell epitopes.**

| **Mouse strain** | **Protein**  **(ORF)** | **SLP sequence *** | **Epitope residues (aa)** | **MHC allele - restriction** | **Stage of expression** | **Inflation?** | **Human CMV Homologue** |
| --- | --- | --- | --- | --- | --- | --- | --- |
| C57BL/6 | M45 | REDVVK**HGIRNASFI**TGCSA | 985-993 | H-2-D^b^ | Early gene | No | UL45 |
| C57BL/6 | M57 | FPACGL**SCLEFWQRV**LQNS | 816-824 | H-2-K^b^ | Early gene | No | UL57 |
| C57BL/6 | m139 | VVLVGARG**TVYGFCLL**SND | 41­9-426 | H-2-K^b^ | Early gene | Yes | US22 |
| C57BL/6 | M38 | VTLI**SSPPMFRV**PVNPVPGG | 316-323 | H-2K^b^ | Early gene | Yes | UL38 |
| C57BL/6 | Altered M38 | EGPPMPMTVTLI**SSPPMFRV** | 316-323 | H-2K^b^ | Early gene | Yes | UL38 |
| C57BL/6 | IE3 | DKSRKYPA**RALEYKNL**PFR | 416-423 | H-2-K^b^ | Immediate early gene | Yes | IE2 |
| C57BL/6 | Altered IE3 | KKCREDKSRKYPA**RALEYKNL** | 416-423 | H-2-K^b^ | Immediate early gene | Yes | IE2 |
| BALB/c | M45 | RITER**VGPALGRGL**YSTVV | 507-515 | H-2-D^d^ | Early gene | No | UL45 |
| BALB/c | m164 | rtwgad**agpprysri**fwav | 257-265 | H-2-D^d^ | Early gene | Yes | - |
| BALB/c | IE1/ pp89 | grlmydm**yphfmptnl**gpsek | 168-176 | H-2-L^d^ | Immediate early gene | Yes | IE1 |

- Bold underlined amino acid (aa) residues within the SLP sequence indicate the CTL epitope.
